# Effect of the GLP-1 Analog Exendin-4 and Oxaliplatin on Intrahepatic Cholangiocarcinoma Cell Line and Mouse Model

**DOI:** 10.3390/ijms141224293

**Published:** 2013-12-13

**Authors:** Ben-Dong Chen, Wen-Chao Zhao, Qing-An Jia, Wen-Yan Zhou, Yang Bu, Zuo-Zheng Wang, Feng Wang, Wu-Jun Wu, Qi Wang

**Affiliations:** 1 Department of Hepatobiliary Surgery, Ningxia Medical University, Yinchuan 750004, Ningxia, China; E-Mails: bdchen6er@21cn.com (B.-D.C.); yangbunxmed@163.com (Y.B.); wangzznxmed@163.com (Z.-Z.W.); wangfnxmed@163.com (F.W.); 2 Department of Hepatobiliary Surgery, Navy General Hospital, Beijing 100086, China; E-Mail: zhaowcngmed@163.com; 3 Department of Hepatobiliary Surgery, Shannxi Province People’s Hospital, Xi’an 710071, Shannxi, China; E-Mails: jiaqasxmed@163.com (Q.-A.J.); wuwjdrsx@163.com (W.-J.W.); 4 Department of ICU, Ningxia Medical University, Yinchuan 750004, Ningxia, China; E-Mail: zhouwynxmed@163.com

**Keywords:** intrahepatic cholangiocarcinoma, GLP-1, exendin-4, mice, PCNA

## Abstract

The influence of Glucagon-like peptide-1 (GLP-1) and Exendin-4 on development of intrahepatic cholangiocarcinoma (ICC) is evaluated in the study. *In vitro* tests, including acute toxicity test, cell colony formation assays, cells proliferation and apoptosis, transwell assay, were performed. An ICC *in situ* tumor animal model was established. Then, animals were randomly divided into four groups (*n* = 6): control, Exendin-4 treatment, oxaliplatin treatment and Exendin-4-oxaliplatin treatment. Animals in the Exendin-4 treatment and Exendin-4-oxaliplatin treatment groups received a subcutaneous injection of Exendin-4 (100 μg/kg/day) for 1 week, and then received oxaliplatin (10 mg/kg/week) by tail vein injection. Animals in the control group received PBS. Immunohistochemistry tests were used for PCNA, Ki67, Caspase 3 expression in tumor tissue. Results show that that, after incubation of human cholangiocarcinoma cell lines, HuCCTI and GLP-1, or HuCCTI and Exendin-4, colony formation number was sharply decreased. However, GLP-1, HuCCTI or Exendin-4 did not affect the colony of normal cells. Combination treatment with oxaliplatin and Exendin-4 can significantly inhibit tumor cells’ proliferation and promote apoptosis. The combined effect is stronger than that of oxaliplatin or Exendin-4. Combination treatment with oxaliplatin and Exendin4 can significantly decrease Ki67 and PCNA proteins’ expression in subcutaneous tumors of nude mice. The inhibitory effect of Combination treatment with oxaliplatin and Exendin4 is clearly stronger than that of oxaliplatin. In addition, Combination treatment with oxaliplatin and Exendin4 can significantly increase Caspase3 protein positive expression. In short, these results show that combination treatment with oxaliplatin and Exendin4 can inhibit tumor cells’ proliferation, and promote apoptosis.

## 1. Introduction

Glucagon-like peptide-1 (GLP-1), an incretin hormone secreted by intestinal L cells, is a promising therapeutic agent in the treatment of diabetes. A major target for GLP-1 actions is the pancreatic β-cell. One of the main physiological roles of this endocrine hormone is to enhance insulin secretion in a glucose-dependent manner [1–3]. The physiological relevance and pharmacology of GLP-1 have been researched extensively, with a major focus on its incretin actions, and its application in the treatment of type-2 diabetes. However, the administration of GLP-1 is not effective as a treatment for diabetes, because the protein is rapidly degraded by dipeptidyl peptidase-4 (DPP-4). Thus, GLP-1 receptor agonists that are resistant to DPP-4 and DPP-4 inhibitors are currently being used for the treatment of type 2 diabetes [4].

Intrahepatic cholangiocarcinoma (ICC) is a devastating malignancy originating from the biliary epithelium, and ranks as the second most common primary liver cancer after hepatocellular carcinoma (HCC) [5]. There are several documented risk factors for ICC, including primary sclerosing cholangitis, liver fluke infection (*Clonorchis sinensis* or *Opisthorchis viverrini*), fibropolycystic liver disease, hepatolithiasis, and thorotrast exposure [6–8]. Exendin-4 is a 39-amino acid glucagon-like peptide-1 (GLP-1) analog, which was found originally in the saliva of the gila monster. It shares approximately 53% sequence homology with the mammalian GLP-1 [9,10]. Because of amino acid changes, exendin-4 is resistant to the degradation of the enzyme dipeptidyl peptidase-4 and has a longer half-life than native GLP-1. Exendin-4 binds to GLP-1 receptors (GLP-1Rs) to exhibit antidiabetic actions, including glucose-dependent stimulation of insulin secretion, delay of gastric emptying, and protection of β-cells.

At present, synthetic Exending-4 has been approved by the FDA for the treatment of diabetes. Other GLP-1 analogs and DDP4 inhibitors were also gradually studied and developed. More and more reports show a close relationship between obesity and diabetes and intrahepatic bile duct cancer [4,6,7]. Inhibiting tumor cell proliferation and promoting tumor cell apoptosis have become the focus of our attention and study. Previous studies have confirmed that GLP-1 can promote normal bile duct cells proliferation and inhibit their apoptosis [4]. However, the effect of GLP-1 on intrahepatic cholangiocarcinoma cells has not been reported. Based on our previous studies [11] about the analysis of clinical data of intrahepatic cholangiocarcinoma and immunohistochemical staining, we find that positive expression of GLP-1R is very high in intrahepatic cholangiocarcinoma tissue, indicating that GLP-1 is closely associated to the occurrence and development of cholangiocarcinoma. Therefore, in the present study, we investigate role of GLP-1 and Exendin-4 in development of intrahepatic cholangiocarcinoma.

## 2. Results

### 2.1. Acute Toxicity Test

**Figure 1.**
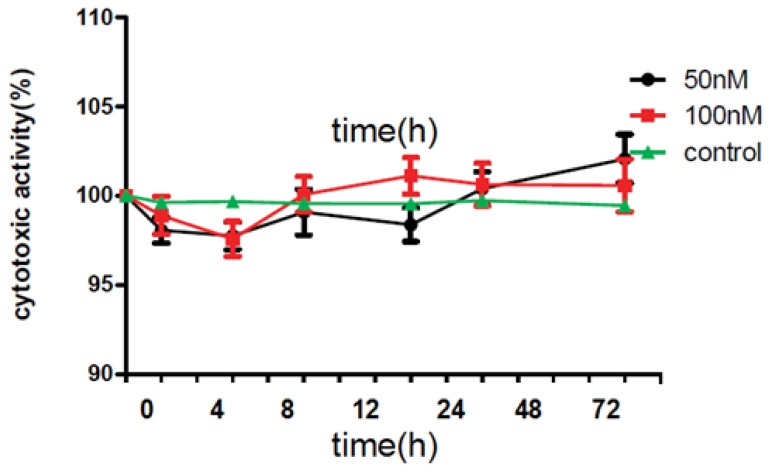
Acute toxicity test.

At time interyals of 4, 8, 12, 24, 48 and 72 h after incubation, HuCCT1 cells were observed in order to check LDH. Results showed that LDH was released in cells. Compared with LDH level in control group, 4, 8, 12, 24, 48 and 72 h after incubation, LDH levels in 50 nM group were 97.34% ± 2.98%, 97.26% ± 1.93%, 98.48% ± 3.64%, 96.72% ± 3.18%, 100.47% ± 2.42%, 101.33% ± 3.81%, respectively. Compared with LDH level in control group, 4, 8, 12, 24, 48 and 72 h after incubation, LDH levels in 100 nM Exendin-4-treated group were 98.72% ± 2.66%, 97.05% ± 2.17%, 99.63% ± 2.44%, 101.72% ± 3.08%, 101.31% ± 3.99%, 99.98% ± 3.63%, respectively (Figure 1).

### 2.2. Cell Colony Formation Assays

**Figure 2.**
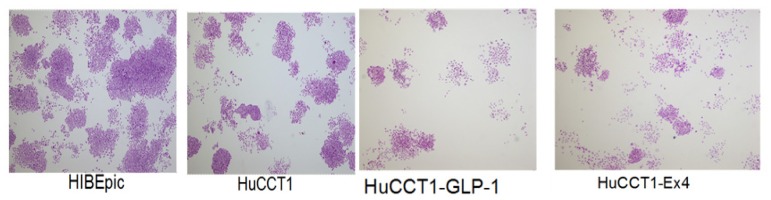
Colony formation experiment.

**Figure 3.**
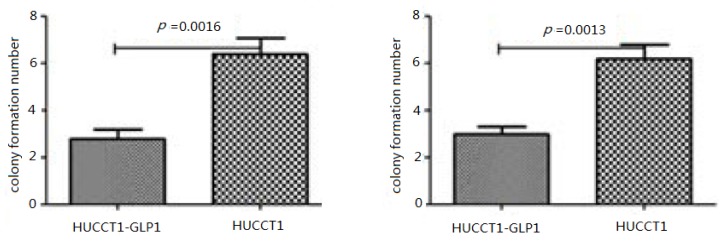
Tumor colony formation after Glucagon-like peptide-1 (GLP-1) and Exendin-4 treatment.

Cell colony formation assays was employed to evaluate effect of GLP-1 and Exendin-4 on HuCCT1 growth. Because GLP-1 could be quickly decomposed by DDP4, after GLP-1 were cultured with cells in serum-free medium for 2 h and then in medium containing serum. In GLP-1 (50 nM) treatment group, colony formation number was sharply decreased (Figures 2 and 3). There was significantly statistical difference between GLP-1-treated group and untreated group (*p* = 0.0016). In Exendin-4 treatment group, colony formation number was sharply decreased. There was significantly statistical difference between Exendin-4-treated group and untreated group (*p* = 0.0013). Effect of Exendin-4 on colony formation efficiency was stronger than that of GLP-1, Exendin-4 was chosen for later experiment. Experimental results showed that after incubation of HuCCTI and GLP-1, or HuCCTI and Exendin-4, colony formation number was sharply decreased. However, GLP-1, HuCCTI or Exendin-4 did not affect the colony of normal cells.

### 2.3. Cells Proliferation

**Figure 4.**
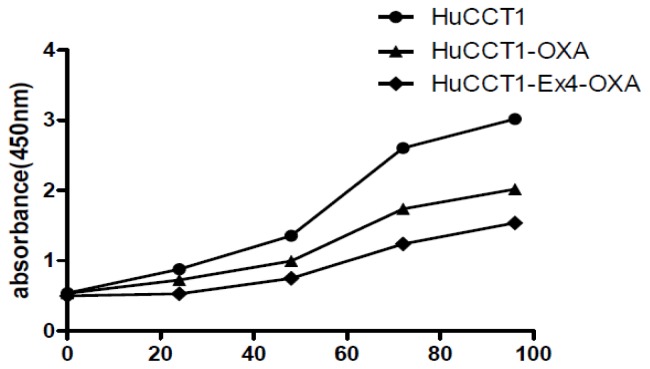
Proliferation rate of intrahepatic cholangiocarcinoma cells treated with oxaliplatin and Exendin-4.

CCK8 dection showed that proliferation rate of intrahepatic cholangiocarcinoma cells treated with oxaliplatin was significantly decreased. Moreover, proliferation rate of intrahepatic cholangiocarcinoma cells treated with oxaliplatin and Exendin-4 was significantly lower than that of intrahepatic cholangiocarcinoma cells treated with oxaliplatin (Figure 4).

### 2.4. Cell Apoptosis

**Figure 5.**
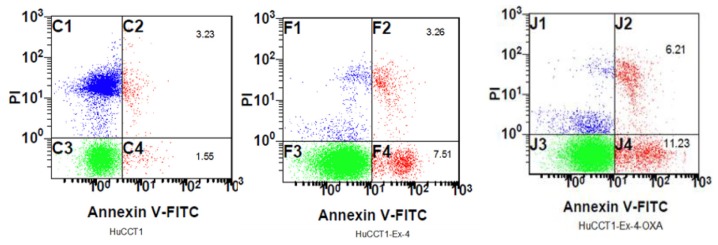
Cell apoptosis was detected by flow cytometry.

The results showed that apoptosis rate of intrahepatic cholangiocarcinoma cells treated with chemotherapy was markedly increased. Moreover, apoptosis rate of intrahepatic cholangiocarcinoma cells treated with chemotherapy was further increased with treatment of Exendin-4. Apoptosis rate was 1.55%, 7.51% and 11.23% (Figure 5). Flow cytometry analysis showed that percent of apoptosis cells were 1.83% ± 0.42%, 7.75% ± 2.00%, 10.76% ± 1.84% in HuCCT1, HuCCT1 + Ex4 and HuCCT1 + Ex4 + OXA groups, respectively (*p* < 0.01).

### 2.5. Transwell Assay Cell Apoptosis

**Figure 6.**
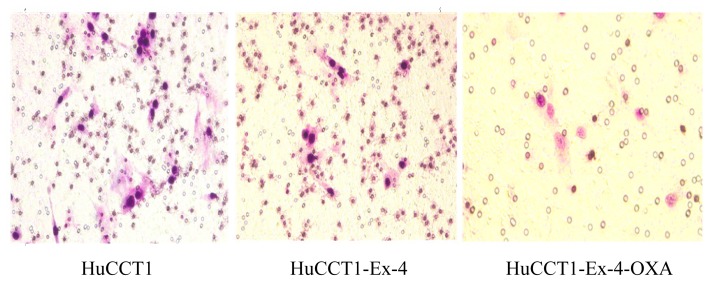
Transwell assay.

Cell migration was slightly delayed by oxaliplatin treatment compared to that in untreated group (HuCCT1-Exendin4 31.61 ± 3.44 *vs*. HuCCT1 43.78 ± 7.66, *p* = 0.0018). Cell migration was significantly delayed by oxaliplatin and Exendin-4 treatment compared to that in the untreated group (HuCCT1-Exendin4-oxaliplatin 15.62 ± 5.52 *vs*. HuCCT1 46.81 ± 8.68, *p* = 0.0003) (Figure 6).

### 2.6. Animal Experiment

Male nude mice (BALB/c), aged 6 weeks, received a subcutaneous injection of 0.5 × 10^6^ HuCCT1 cells (100 μL 1:1 PBS). Animals were randomly divided into four groups (*n* = 6): control, Exendin-4 treatment, oxaliplatin treatment and Exendin-4-oxaliplatin treatment. Animals in Exendin-4 treatment and Exendin-4-oxaliplatin treatment groups received a subcutaneous injection of Exendin-4 (100 μg/kg/day) for 1 week, and then received oxaliplatin (10 mg/kg/week) by tail vein injection. Animals in control group received PBS. Tumor size was measured every week. Tumor size *V* = 1/2 × *a* × *b*^2^. After 5 weeks, animals were killed and weighed.

**Figure 7.**
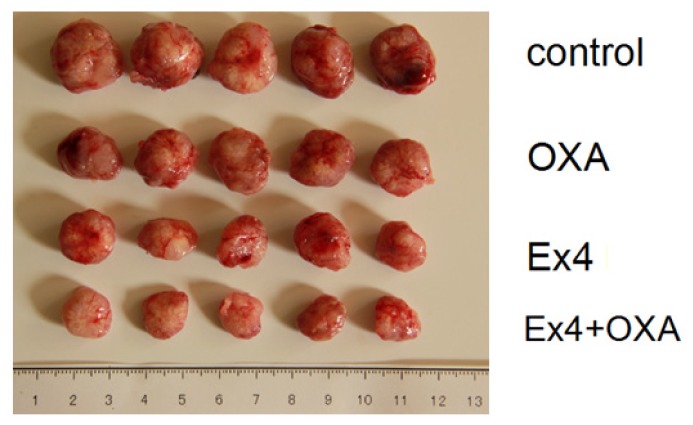
Nude mice bearing tumor.

**Figure 8.**
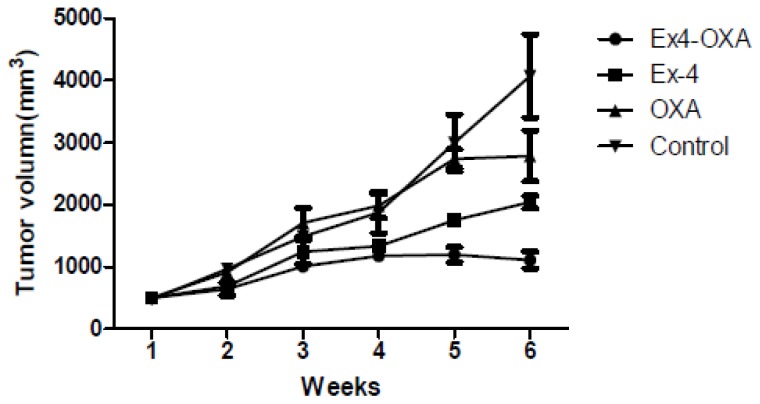
Tumor volume in different groups.

Results showed that mean tumor size was 1742, 1117 and 940 mm^3^ in oxaliplatin treatment, Exendin-4 treatment and Exendin-4-oxaliplatin treatment, respectively. There was significantly statistical difference between treatment groups and control group (*p* = 0.0104) (Figures 7 and 8).

Experiment results showed that mean tumor size (2117 mm^3^) in the control group was the biggest in the four groups. Mean tumor size was the smallest in the Exendin4 + oxaliplatin group. The result was in agreement with that in cell culture test. Namely, Exendin4 could increase the sensitivity of chemotherapy drugs. In addition, Exendin4 might still inhibit tumor cells’ proliferation.

**Figure 9.**
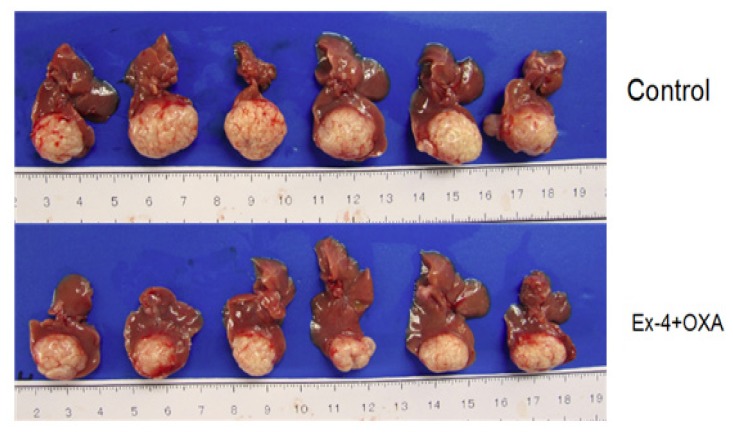
Tumor size in different groups.

HuCCT1 tumor size (control group 2.81 ± 0.25 cm^3^*vs*. Exendin-4 + oxaliplatin group 1.89 ± 0.42 cm^3^, *p* = 0.0104) was significantly decreased after oxaliplatin (10 mg/kg/week) and Exendin-4 (100 μg/kg/day) (Figure 9).

**Figure 10.**
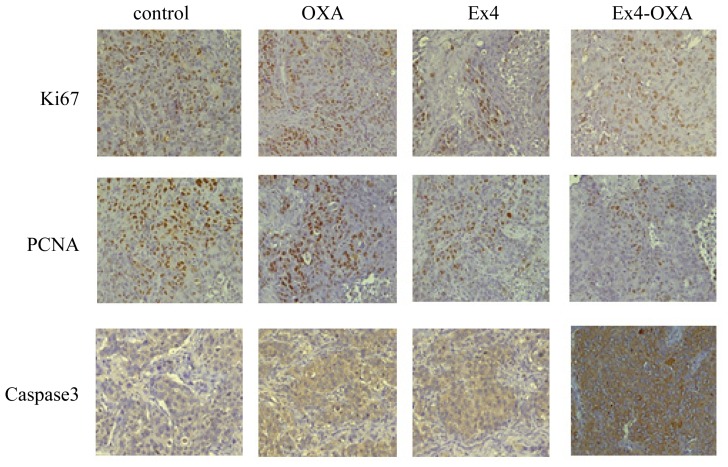
Tumor proliferation and apoptosis in different groups.

Immunohistochemical analysis showed that the positive expression rate of proliferation associated antibody Ki67 and PCNA in tumor was markedly increased. Positive expression rate of proliferation associated antibody Ki67 and PCNA in mice treated with Exendin4 and oxaliplatin was significantly weaker than that in mice treated with oxaliplatin. Apoptosis associated antibody Caspase3 in mice treated with Exendin4 and oxaliplatin displayed strong positive expression. The result indicated that combination of Exendin4 and oxaliplatin could have a stronger inhibitory effect on tumor proliferation and promotive effect on apoptosis (Figure 10).

## 3. Discussion

In the present study, we found that effect of Exendin4 on intrahepatic cholangiocarcinoma cell line HuCCT1 proliferation is obvious. In acute cells toxicity test of drug, we confirm that Exendin-4 has no acute toxic effect and can be applied to cell culture. We found that oxaliplatin can inhibit tumor cells’ growth, but promote normal cell proliferation. Exendin4 can increase the sensitivity of intrahepatic cholangiocarcinoma cells to chemotherapy drug oxaliplatin. CCK8 test confirms that combination of oxaliplatin and Exendin-4 can decrease tumor cell activity to 8%. Invasion and Migration Experiments show that oxaliplatin or Exendin-4 treatment does not obviously affect HuCCT1 cells’ transmembrane capability. There is no statistical difference in HuCCT1 cells’ transmembrane capability between oxaliplatin-treated group and control group, or between Exendin-4 treated group and control group. However, combination treatment with oxaliplatin and Exendin-4 can greatly decrease HuCCT1 cells transmembrane capability. This further confirms that Exendin4 can increase the sensitivity of intrahepatic cholangiocarcinoma cells to chemotherapy drug oxaliplatin. In addition, Western blot analysis show that PCNA protein expression downregulate, and Caspase 3 protein expression upregulate, indicating that tumor cells’ proliferation was significantly inhibited by combination treatment with oxaliplatin or Exendin-4, but apoptosis rate increased. This is in agreement with Hagai Ligumsky’s research about breast cancer [12]. Animal experiment also obtains similar results. Oxaliplatin had no obvious inhibitory effect on subcutaneous tumor of nude mice. Exendin4 had obvious inhibitory effect on subcutaneous tumor of nude mice. Moreover, combination treatment with oxaliplatin and Exendin4 can obviously decrease subcutaneous tumor size of nude mice. Combination treatment with oxaliplatin and Exendin4 also obtain similar results to liver orthotopic implantation tumor in nude mice. Combination treatment with oxaliplatin and Exendin4 can significantly decrease Ki67 and PCNA proteins expression in subcutaneous tumor of nude mice. Inhibitory effect of Combination treatment with oxaliplatin and Exendin4 is obviously stronger than that of oxaliplatin. In addition, Combination treatment with oxaliplatin and Exendin4 can significantly increase Caspase3 protein positive expression. These results show that combination treatment with Oxaliplatin and Exendin4 can inhibit tumor cells proliferation, and promote apoptosis.

Marco Marzioni reported GLP-1R gene expression in bile duct cells and its effect on bile duct cells. Moreover, in the case of cholestasis [13], GLP-1 is necessary to reactive hyperplasia of bile duct cells; Bile duct cells are extremely sensitive to the activation of GLP-1R. Another study shows that analogs of GLP-1, Exendin-4, can effectively inhibit the bile duct cells apoptosis induced by glycochenodeoxycholic acid *in vitro*, and bile duct cell apoptosis in rats with bile duct ligation induced by carbon tetrachloride [14]. Therefore, Exendin4 can promote normal bile duct hyperblastosis, and inhibit apoptosis. Our current work found that combination treatment with oxaliplatin and Exendin4 can inhibit tumor cells’ proliferation, and promote apoptosis. This is consistent with Hagai Ligumsky’s research about breast cancer [12]. Paolo Onori *et al*. found that secretin can inhibit bile duct cancer cell growth by regulating secretin receptor cAMP dependent signal pathway. Secretin can restore cell cytoactivity by inhibiting exendin-4 induced cAMP activation [15]. This indicates that cAMP might be the main reason for the antitumor activity of exendin-4. Effect of GLP-1 on beta cell function is mediated by GPCR. GLP-1 may activate protein kinase C, MAPK, ERK1/2, AKT, and CREB by increasing cAMP expression. CAMP and its inhibitors 8-Cl-cAMP induce tumor cells’ apoptosis, enhance p21, p27 levels, inhibit breast cancer, prostate cancer, ovarian cancer and colon cancer growth. GLP-1 and exendin-4 can enhance cAMP level in bile duct carcinoma cells. The growth inhibitory activity of exendin can was reversed by reducing the cAMP level. This suggests that upregulation of cAMP may induce inhibitory effect of exendin-4 against tumor cell growth. p38 MAPK is a potential related growth target gene of cAMP, and it can induce growth arrest or apoptosis.

When Exendin-4 receptor exposed to high concentrations of ligand, saturation and desensitization phenomenon occur up. These results in the activation of cAMP, inhibition of tumor proliferation, promote apoptosis of tumor cells. GLP-1 can still activate β-arrestin signal pathway, promote pro-apoptotic protein Bad inactivation and inhibition of apoptosis. GLP-1 is a potent inducer of cAMP. It can inhibit intrahepatic cholangiocarcinoma cells’ proliferation. Downregulation of GLPL-1 gene expression might be a new mechanism of intrahepatic hepatocellular carcinoma development in obesity and diabetes patients.

## 4. Experimental Section

### 4.1. Cell Culture

The HuCC-T1 and HIBEpic cells were grown in suspension in RPMI 1640 medium (Gibco BRL, Grand Island, NY, USA) supplemented with 15% fetal-calf serum (Hyclone, Logan, UT, USA), penicillin (100 mg/mL), streptomycin (100 mg/mL), and 2 mM l-glutamine (Gibco BRL, Grand island, NY, USA). Cells were maintained at 37 °C in an atmosphere of 5% carbon dioxide and 95% air and underwent passage twice weekly.

### 4.2. Cell Toxicity Test

HuCC-T1 cells were cultured in 6-well plates for 24 h. Then, cell suspended in 1 mL double-strength Dulbecco’s modified Eagle’s medium (DMEM) supplemented with 20% fetal bovine serum (FBS) and 4 mM l-glutamine, which had been mixed with 1 mL 0.6% agarose and then held at 60 °C. Cells were divided into four groups: background control (inculbation in medium), low controls (cells spontaneously release LDH), high controls (LDH was maximumly released when the cell membrane ruptures), and experimental substance (medium containing 2 mg/mL or 4 mg/mL GLP-1R or Exendin-4). Values were measured at 492 nm (OD492) after cells were cultured for 4, 8, 12, 24, 48 or 72 h.

(1) Cell toxicity ratio=(experimental value-low control)/(high control-low control)×100%

### 4.3. Cloning Formation

HuCC-T1 cells were cultured in 6-well plates for 24 h. Colonies were counted after 3 weeks.

### 4.4. Flow Cytometric Analysis of Apoptosis

Apoptosis was quantified by a flow cytometry (MoFlo FACS, Dako, Glostrup, Denmark). Cells were cultured in medium containing oxaliplatin for 48 h. Then, cells were centrifuged (300× *g*, 10 min, 4 °C) and washed with 5 mL PBS containing glycine (2%, *w*/*v*) and bovine serum albumin (0.1%, *w*/*v*). The detection of apoptotic cells was performed with the *In Situ* Cell Death Detection Kit (TMR Red, Roche Diagnostics, Mannheim, Germany).

### 4.5. Transwell Migration Assay

The migration of UCMSCs was measured using Transwell plates (Corning Costar, Tewksbury, MA, USA) with 8 μm pore filters, as previously described by Kim *et al*. [10].

### 4.6. Animals and ICC Nude Mice with Subcutaneous Tumor Model

Male nude mice (BALB/c), aged 4–6 weeks, received a subcutaneous injection of 0.5 × 10^6^ HuCCT1 cells (100 μL 1:1 PBS). After 1 week, tumors were formed. Animals were randomly divided into four groups (*n* = 6): control, Exendin-4 treatment, oxaliplatin treatment and Exendin-4-oxaliplatin treatment. Animals in Exendin-4 treatment and Exendin-4-Oxaliplatin treatment groups received a subcutaneous injection of Exendin-4 (100 μg/kg/day) for 1 week, and then received oxaliplatin (10 mg/kg/week) by tail vein injection. Animals in control group received PBS. Tumor size was measured every week. Tumor size *V* = 1/2 × *a* × *b*^2^. After 5 weeks, animals were killed and weighed.

### 4.7. Establishment of ICC *in Situ* Tumor Animal Model

Male nude mice (BALB/c), aged 4–6 weeks, received a subcutaneous injection of 0.5 × 10^6^ HuCCT1 cells (100 μL 1:1 PBS). After 3 weeks, mice were killed and tumors were removed. Then, tumors were cut into small pieces (2 mm × 1 mm × 1 mm).

Twenty-four animals were anaesthetized. The left costal margin was incised and liver was exposed. Tumor pieces were implanted into livers and incision was closed. Then, animals were randomly divided into four groups (*n* = 6): control, Exendin-4 treatment, oxaliplatin treatment and Exendin-4-oxaliplatin treatment. Animals in Exendin-4 treatment and Exendin-4-oxaliplatin treatment groups received a subcutaneous injection of Exendin-4 (100 μg/kg/day) for 1 week, and then received oxaliplatin (10 mg/kg/week) by tail vein injection. Animals in control group received PBS. Tumor size was measured every week. Tumor size *V* = 1/2 × *a* × *b*^2^. After 5 weeks, animals were killed and weighed.

### 4.8. Immunohistochemistry Test for PCNA, Ki67, Caspase 3 Expression in Tumor Tissue

Five micrometer-thick sections of paraffin-embedded tissues were deparaffinized, heated in citrate buffer (0.01 M, pH 6.0) in a microwave for 3 min, and then treated with 0.3% hydrogen peroxide in methyl alcohol for 20 min to block endogenous peroxidase activity. After three washes in PBS, the sections were incubated with 10% normal goat serum (Vector ABC Elite Kit; Vector Laboratories, Burlingame, CA, USA) and then incubated for 1 h at room temperature with primary antibody. As a negative control, primary antibody was omitted. After three washes in PBS, the sections were treated with the appropriate secondary antibody (dilution 1:200; Vector Laboratories, Burlingame, CA, USA) for 45 min, washed three times in PBS, and then incubated for 45 min with avidin–biotin peroxidase complex (Vector ABC Elite kit, Burlingame, CA, USA), prepared according to the manufacturer’s instructions. The peroxidase was reacted using a diaminobenzidine substrate kit (Vector Laboratories, Burlingame, CA, USA). The sections were counterstained with hematoxylin, dehydrated, and cleared with xylene before finally being mounted.

### 4.9. Statistical Analysis

Statistical analysis was carried out using SPSS version 10.0 for Windows software (SPSS, Chicago, IL, USA). As all groups showed normal distribution, group differences were analyzed using parametric statistical methods, paired independent sample *t*-tests following one-way ANOVA. Data were presented as mean ± standard deviation, *p* < 0.05 was considered statistically significant.

## 5. Conclusions

Proliferation of tumor cells treated with GLP-1 or Exendin4 were significantly inhibited. In addition, apoptosis of tumor cells treated with oxaliplatin or Exendin4 were also significantly increased. Moreover, combined effect of Exendin-4 with oxaliplatin show stronger effect in promoting tumor cells’ apoptosis. Combination of Exendin-4 with oxaliplatin also significantly inhibited tumor cells’ invasion and migration. Animal experiment showed that the proliferation associated antibodies, Ki67 and PCNA, display high positive expression in tumor tissue, which are decreased by Exendin4 and oxaliplatin. Caspase3 display strong positive expression in tumor tissue treated with Exendin4 and oxaliplatin. Combination of Exendin4 and oxaliplatin can significantly inhibit tumor cells’ proliferation and tumor growth, promote apoptosis, and display strong antitumor activity.
